# A Micro-Computed Tomography Analysis of Void Formation in Apical Plugs Created with Calcium Silicate-Based Materials Using Various Application Techniques in 3D-Printed Simulated Immature Teeth

**DOI:** 10.3390/dj13090385

**Published:** 2025-08-25

**Authors:** Krasimir Hristov, Ralitsa Bogovska-Gigova

**Affiliations:** Department of Pediatric Dentistry, Faculty of Dental Medicine, Medical University of Sofia, 1000 Sofia, Bulgaria; r.bogovska@fdm.mu-sofia.bg

**Keywords:** immature teeth, apical plug, bioceramic putty, voids formation, micro-CT

## Abstract

**Background/Objectives**: The management of immature teeth with wide apical foramina presents significant challenges in endodontic treatment due to difficulties in achieving a hermetic seal. The aim of this study was to evaluate void formation in apical plugs created using three calcium silicate-based materials—Biodentine, NuSmile NeoPUTTY, and Well-Root PT—applied with the help of manual, ultrasonic, or rotary file condensation (XP-endo Shaper) in 3D-printed immature teeth. **Methods**: Micro-computed tomography analysis was used to assess the internal, external, and total void percentage of material volume. The statistical analysis was performed using two-way ANOVA and the post hoc Bonferroni test. Statistical significance was set at *p* < 0.05. **Results**: The materials and techniques used individually do not significantly influence the formation of internal voids, but their combination does (F(4, 99) = 2.717, *p* = 0.034). Both factors and their interaction are significant for external voids (F(4, 99) = 4.169, *p* = 0.004), and all have a notable effect on total void percentages (F(4, 99) = 3.456, *p* = 0.012). No significant differences were observed in internal voids across the groups (*p* > 0.05), ranging from 0.635% to 1.078%. External voids varied significantly, with Well-Root PT and ultrasonic condensation showing the highest values with a significant difference (*p* < 0.05), while NeoPUTTY and Biodentine with XP-endo Shaper exhibited the lowest. Total voids remained below 4%, with no significant differences among manual condensation groups. Neither material type nor application technique consistently influenced void formation, except for Well-Root PT with ultrasonic condensation. **Conclusions**: These findings suggest that modern bioceramic materials and application techniques produce comparable, low-void apical plugs, with XP-endo Shaper showing promise for minimizing external voids. The interaction between material and application technique plays a crucial role during the creation of apical plugs.

## 1. Introduction

Teeth with pulp necrosis and immature apices, commonly encountered in young patients due to traumatic injuries or extensive caries, pose significant challenges in endodontic management [1]. They exhibit wide apical foramina, thin dentinal walls, and incomplete root development, which complicates conventional root canal treatment [2]. The absence of a definitive apical stop—a natural narrowing of the root canal at the apex—makes it difficult to achieve a hermetic seal during obturation, increasing the risk of over-extrusion of filling materials into periapical tissues or inadequate sealing, both of which can lead to persistent infection or treatment failure [3,4]. In contrast to a tooth with a mature apex, which can be treated with standard procedures without special requirements, an immature tooth with pulp necrosis requires more time-consuming and complex therapy. One of the methods historically used for this situation is apexification with the placement of Ca(OH)_2_ paste into the canal to promote the formation of a calcific barrier at the root apex. This technique requires repeated and extended dressing of the root canal, which can range from 5 to 20 months (with an average duration of 12.9 months) [5]. The extended and variable duration of calcium hydroxide apexification may lead to a decrease in patient compliance and has been associated with a higher risk of bacterial contamination due to microleakage in temporary restorations [6]. Additionally, this treatment approach leads to potential weakening of the root, resulting in a reduction in fracture resistance of the tooth [7].

To address these challenges, endodontic management of immature apices involves the creation of an artificial apical barrier using a number of new specialized materials that have emerged onto the commercial market in recent years [3,8]. Among the most commonly used materials are calcium silicate-based cements, Biodentine, and other bioceramic compounds [9,10]. These materials are selected for their biocompatibility, bioactivity, and ability to promote hard tissue formation, which is critical for long-term success in these cases [11].

MTA was among the first calcium silicate cements, used in endodontic treatment for immature teeth, and is considered a gold standard [12]. It is composed primarily of tricalcium silicate, dicalcium silicate, and bismuth oxide, contributing to its excellent sealing properties and ability to stimulate dentinogenesis and/or cementogenesis upon contact with periapical tissues [13]. However, it has limitations such as handling difficulties, longer setting times, and the possibility of discoloration of the remaining tooth structure [14]. Biodentine is a calcium silicate-based material that provides similar benefits but has a faster setting time and better handling properties, making it a favored choice for apical sealing among clinicians [15].

Nowadays, premixed bioceramic putties are gaining popularity in contemporary dental practices. These materials are either a paste in a syringe or putty in a jar, ready for application. Zirconium and tantalum oxide serve as radiopacifying agents, replacing the bismuth oxide traditionally used in MTA [9]. Consequently, they are hydrophilic and do not set within their packaging but harden upon contact with moisture when applied, preventing tooth discoloration [16]. Furthermore, these materials exhibit properties such as biocompatibility, insolubility, dimensional stability, and high alkalinity [14].

The evaluation of physical parameters, such as void formation in apical plugs, is critical for assessing the sealing quality of endodontic materials in teeth with immature apices. To this end, 3D-printed plastic models of immature teeth provide a standardized and reproducible experimental platform that simulates key anatomical features, such as wide apical foramina and divergent root walls. This controlled environment facilitates precise micro-computed tomography analysis of internal and external voids, enabling reliable comparisons of material performance and application techniques. The use of 3D-printed models has been increasingly adopted in endodontic research to overcome the variability of natural teeth, particularly for complex anatomies, ensuring consistent canal morphology and enhancing the reproducibility of results [17]. By focusing on physical characteristics, this study aims to optimize apexification procedures by evaluating the void formation of calcium silicate-based materials under various condensation techniques in 3D-printed immature teeth.

NeoPUTTY (NuSmile Ltd., Houston, TX, USA) is a premixed calcium silicate-based bioceramic material intended for endodontic filling and apical obturation in immature permanent teeth. Studies have shown that NeoPUTTY exhibits acceptable biocompatibility and promotes mineralization, similar to conventional materials such as MTA and NeoMTA Plus, without significant cytotoxicity to human pulp cells [18,19,20].

Well-Root PT™ (Vericom, Chuncheon-si, South Korea) is a bioceramic paste with proven clinical and laboratory applicability as an apical sealing material for teeth with incomplete root development. Scientific studies have shown that Well-Root PT exhibits high biocompatibility [21]. In vitro and clinical data show cell viability and mineralization potential comparable to or exceeding those of MTA and other bioceramic materials, such as NuSmile NeoPUTTY. Well-Root PT achieves similar clinical and radiographic success rates to MTA, and it has additional advantages such as easier clinical use and application and no tooth discoloration [22,23]. In terms of hermetic sealing ability, Well-Root PT and other bioceramic materials demonstrate high radiopacity and microhardness similar to or better than MTA, supporting their use as apical filling materials [22].

Bioceramic materials are commonly used to create a 3–5 mm thick, impermeable apical plug in the root canal. This artificial barrier helps facilitate the subsequent obturation of the remaining space in the canal [23]. During the endodontic treatment of immature teeth, after the root canal has been cleaned, shaped, and disinfected, the cement is carefully placed in the apical area. This is often conducted with the help of a special carrier or hand instrument, and the material is compacted to ensure even distribution. Once the material sets, it forms a biocompatible barrier that not only seals the apex of the tooth but also promotes the formation of a calcium bridge, which further strengthens the plug over time [24].

The main techniques for creating an apical plug in necrotic immature teeth are manual, ultrasonic, or condensation with a rotary endodontic file [25]. Each method affects the formation of voids in the material and its ability to seal hermetically differently. Manual condensation is a traditional technique using pluggers or gutta-percha points [25]. It is easy to use but it increases the risk of forming defects and/or voids in the material and poorer adaptation to the root canal walls, especially in complex anatomical configurations and curved canals. However, some studies have found that precise manual condensation can ensure good adaptation and minimal defects, especially when using MTA [26,27].

Ultrasonic vibration entails applying ultrasonic energy to the material or instrument during the placement of the apical plug [25]. This technique reduces internal porosity and improves adaptation to the dentinal walls [28]. However, excessive ultrasonic vibration (greater than 2–8 s per step) can increase porosity and reduce the microhardness of the material [29,30]. Ultrasonic activation is beneficial when using MTA and other cements, but its effect on premixed bioceramic materials such as NuSmile NeoPUTTY and Well-Root PT is not well-studied.

Condensation with a rotary endodontic file is also intended to improve the fluidity of the material and, consequently, its adaptation. The general principle is that this condensation can improve the seal of bioceramic materials, potentially reducing void formation [31].

External and internal voids are commonly found in apical plugs. The presence of these voids negatively impacts the quality and sealing ability of the apical plug. Open (external) voids are particularly problematic as they are associated with increased microleakage, providing a pathway for bacterial invasion, and reducing the long-term success of apexification procedures [32]. Although less common, internal voids can also compromise the structural integrity of the plug [33]. Additional research is needed to evaluate the formation of external and internal voids during the creation of apical plugs in teeth with incomplete root development, using modern bioceramic materials and various condensation techniques.

The aim of the present study was to assess the external and internal voids of three calcium silicate cements (Biodentine, NuSmile NeoPUTTY, and Well-Root PT) applied using three different techniques: manual condensation, ultrasonic condensation, and rotary endodontic file condensation, when creating apical plugs in immature teeth. The null hypothesis stated that there would be no statistically significant differences in void formation among the various experimental materials and compaction techniques following the creation of the apical plug.

## 2. Materials and Methods

### 2.1. Sample Size Calculation

Based on previous studies from the specialized literature [14,31], and calculations performed with G-Power statistical power analysis software (version 3.1.9.7), a total sample size of n = 108, with 12 teeth per group, was determined to be sufficient for detecting a large effect size (W = 0.48) with a statistical power of 0.95 (95%) and a significance level of 0.05 (5%) for a hypothesis test.

### 2.2. Tested Materials

Table 1 presents the study materials.

For this study, standardized, 3D-printed upper first incisors with prepared access cavities and measuring 21 mm in length were used (DRSK, Hässleholm, Sweden). These models simulated incomplete root development, featuring divergent apical root walls (blunderbuss apex) and had a canal diameter of 2 mm at the apical part. The study included a total of 108 teeth, divided into nine groups of 12 based on the application method and the type of apical sealing material used. A single clinician performed all procedures. Cements were introduced into the canal, and apical plugs were created using a microscope (Zumax OMS1800, Zumax Medical, Suzhou, China). For manual and ultrasonic condensation techniques, the plugger was selected based on its ability to fit snugly within the standardized 3D-printed root canal, which had a consistent apical diameter of 2 mm. Specifically, a Dentsply Sirona (Ballaigues, Switzerland) endodontic plugger (Schilder Plugger Anterior №11A, 1.0 mm tip diameter) was chosen, as its diameter was approximately 50% of the canal’s apical diameter, ensuring effective condensation without excessive force that could disrupt the material or canal walls. This size was verified under a microscope to confirm a close fit to the canal walls while allowing controlled compaction of the apical plug. For manual condensation, the plugger was used to incrementally compact the material to the desired thickness. For indirect ultrasonic condensation, the same plugger was employed. The standardized canal dimensions of the 3D-printed models ensured consistent plugger fit across all samples, minimizing variability in condensation outcomes.

For the indirect ultrasonic condensation groups, a 10 s duration of ultrasonic activation at 25 kHz was selected to optimize material flow and adaptation of the bioceramic cements to the canal walls, ensuring uniform compaction of the apical plug while minimizing the risk of material displacement. Previous studies have suggested limiting ultrasonic activation to 8 s to avoid increased porosity or reduced microhardness in materials like MTA [30]. However, the premixed nature and distinct rheological properties of the tested bioceramics, combined with the standardized canal geometry of the 3D-printed models, supported a slightly extended duration to achieve effective condensation without compromising material integrity. The 10 s application was performed under microscopic visualization to ensure controlled energy delivery.

For Groups 2, 5, and 8, condensation was performed using the XP-endo Shaper (XPS) (FKG Dentaire SA, La Chaux-de-Fonds, Switzerland), operated at a rotational speed of 800 rpm and a torque of 1 N.cm, as recommended by the manufacturer for optimal performance in wide canals. The XPS was positioned 1 mm shorter than the working length and activated for 10 s in a counterclockwise motion, with the instrument retracted while maintaining rotary motion to ensure uniform material distribution [31].

The plugs were compacted with the fitted pluggers (Dentsply Sirona, Germany) to a 3 mm thickness in the apical region. Excess apical material was removed with a sterile blade if overfilling occurred. All samples were stored in an incubator at 37 °C with 100% relative humidity for one week to ensure complete setting and simulate internal oral conditions.

The groups and methods of application are summarized in Table 2.

### 2.3. Scanning Procedure

To measure the external, internal, and total voids and gaps in the apical plug, the teeth were scanned using a high-resolution desktop micro-CT scanner (SkyScan 1272, SkyScan, Bruker, Kontich, Belgium). The plastic samples from all groups were positioned in a mold to maintain consistent alignment during scanning. The scanning parameters were set as follows: a voltage of 85 kV, a current of 117 μA, and a 1.0 mm aluminum filter. The pixel size was 9 μm, with a rotation step of 0.9° and a complete 360° sample rotation. Frame averaging was set to 4, with an exposure time of 140 ms per projection. The average scan duration per sample was approximately 9 min. The NRecon software (version 2.2.0.6, Bruker, Kontich, Belgium) was used to reconstruct the tooth projections into cross-sectional slices, applying the following settings: ring artifact correction and smoothing at 0, beam hardening correction at 55%, and adjustments for misalignment compensation.

### 2.4. Evaluation of Internal and External Voids in the Samples Using Micro-CT

The analysis was performed after reconstructing the specimens with CTAn visualization software (Bruker, Kontich, Belgium), version 1.23.01. This process involved assessing the 3D microarchitecture of each apical plug. Global thresholding was applied by visual matching with grayscale images. The same global threshold values were applied to all samples from the same groups. Distinct segmentation was used to differentiate voids from the materials of the apical plugs, based on a micro-CT resolution of 9 μm. The binarization protocol involved manual verification of segmented images against original scans to confirm segmentation accuracy, minimizing artifacts from the radiopaque materials.

External voids were defined as gaps at the surface of the apical plug material. To differentiate between the apical plug (comprising radiopaque bioceramic materials) and the tooth walls, which appear dark in certain grayscale micro-CT images, a combination of global thresholding and distinct segmentation techniques was employed. The radiopaque bioceramic materials (containing zirconium and tantalum oxide) exhibited higher radiodensity compared to the plastic tooth walls, allowing for clear differentiation during segmentation. The thresholding process was optimized by visual matching with grayscale images, and manual verification of segmented images against original scans was performed to ensure accuracy. This process minimized misidentification of artifacts and confirmed the delineation of voids as gaps with lower radiodensity at the interface.

Internal voids were identified as air pockets or gaps completely enclosed within the material, while external voids were defined as gaps at the interface between the apical plug and dentinal walls. Total voids were the sum of internal and external voids. The volumes of both internal and external voids were expressed as a percentage (%) of the total material volume.

### 2.5. Statistical Analysis

The data followed a normal distribution (Shapiro-Wilk test, *p* > 0.05) with homogeneous variances (Levene’s test, *p* > 0.05). A two-way ANOVA analysis and the post hoc Bonferroni test were conducted to assess the effect of apical plug material and application technique on the percentage of formed voids. Statistical analyses were conducted with SPSS, Version 19.0 (IBM Corp., Armonk, NY, USA). Differences among experimental groups were deemed significant at *p* < 0.05.

## 3. Results

**Figure 1:**
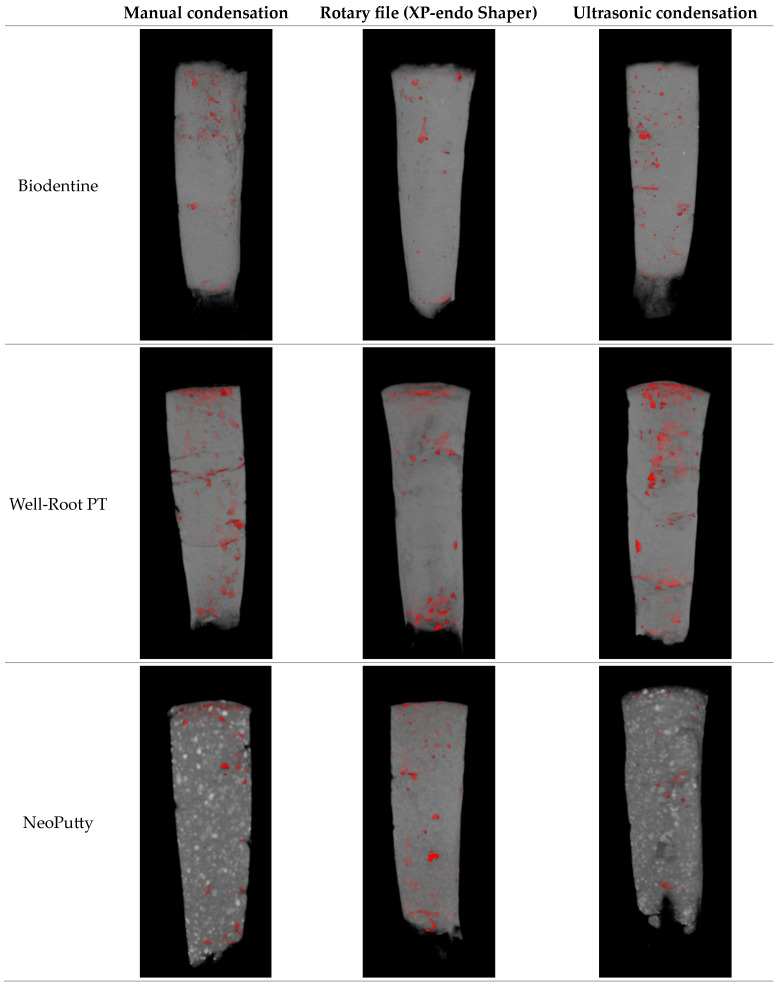
Representative micro-CT images of apical plugs, created with different materials and condensation techniques with superimposed voids (red color).

Figure 1 presents the micro-CT images of the studied groups.

A two-way ANOVA was conducted to examine the effect of materials, application technique, and their interaction on the dependent variables internal, external, and total voids (Table 3).

The results indicate that while the materials and application technique individually do not have a significant effect on the percentage of internal voids, their interaction does (F(4, 99) = 2.717, *p* = 0.034). This suggests that the combination of specific materials and techniques can influence the formation of internal voids in the apical plugs. On the other hand, both material and technique have significant main effects on external voids, and their interaction further amplifies this effect (F(4, 99) = 4.169, *p* = 0.004). A similar pattern was observed in the analysis of total voids, where the main effects of materials and application techniques demonstrated a statistically significant influence on void percentage. The analysis of the interaction effect indicates that certain combinations of materials and techniques may lead to a higher or lower total percentage of voids than would be expected if considering each factor independently (F(4, 99) = 3.456, *p* = 0.012).

Table 4 presents the percentage of external, internal, and total voids within the material volume in all studied groups.

No significant differences were found in the percentage of internal voids (*p* > 0.05). Their mean values ranged between 0.64% and 1.08%. Similar homogeneity between materials and techniques was observed, probably due to their calcium silicate-based composition. Differences existed in the percentage of external voids. Group 6 (Well-Root PT and ultrasonic condensation) had the highest values, and the comparison with the other groups showed statistically significant differences (*p* < 0.05). The NeoPutty and Biodentine groups applied with XP-endo Shaper had the lowest percentage of external voids. Regarding the total percentage of voids formed, there were no significant differences between the manual condensation groups (1.46–2.44%) (*p* > 0.05). The data obtained from all experimental groups showed the existence of external and internal voids in all materials studied, indicating that almost no combination of material-application techniques was superior to the others. In all groups, the total percentage of voids did not exceed 4% of the apical plug volume.

## 4. Discussion

The present study examined void formation during the apical plug creation using various techniques in immature teeth. It was found that neither the application technique nor the type of material significantly affected the quality of the obturation, except when ultrasonic vibrations were used in combination with WellRoot PT. This led to the formation of a higher percentage of external voids. As a result, the null hypothesis was partially accepted as valid. Current techniques indicate that achieving root canal filling without any voids or defects is not possible. Factors such as the operator’s experience, the quality of root canal procedures, spreader paths, the properties of the sealer, and its application method can all impact the outcome [34,35,36]. The absence of voids in root canal obturation has been shown to significantly improve treatment success [37].

The use of 3D-printed plastic models in this study provided several advantages for evaluating void formation in apical plugs, as they ensured standardized canal dimensions and eliminated anatomical variability inherent in natural teeth, facilitating reliable comparisons across groups. This standardization allows for precise micro-computed tomography analysis of physical parameters like void formation, minimizing confounding factors such as irregular canal morphology or dentinal tubule moisture [13,14]. However, these models lack the biological properties of natural teeth. Natural teeth, while more representative of clinical conditions, pose challenges in endodontic studies due to their anatomical variability, which complicates group homogeneity and reproducibility [10]. Thus, 3D-printed models were chosen to ensure experimental control, but future studies in natural teeth are needed to validate these findings under biological conditions.

The low-void percentages observed in this study, not exceeding 4% across all groups, underscore the efficacy of Biodentine, NuSmile NeoPUTTY, and Well-Root PT as apical plug materials in 3D-printed models, consistent with recent micro-CT studies evaluating bioceramic materials in endodontic applications. Eldehna et al. (2025) reported void percentages ranging from 3.73% (NeoMTA Putty) to 9.28% (Bio-C Repair) in natural teeth, attributing differences to material consistency and dentinal fluid interactions that enhance hydration and sealing in vivo [14]. Similarly, Drukteinis utilized micro-CT to assess bioceramic sealers in root canal obturation, noting that material flowability and hydration significantly influence void formation and adaptation to canal walls [38]. Furthermore, Drukteinis et al. investigated the effect of direct ultrasonic agitation on BioRoot RCS and MTA Flow in apically perforated roots, finding significantly higher porosity in MTA Flow fillings, particularly with ultrasonic agitation, due to excessive vibratory forces potentially increasing air incorporation [38]. These findings align with our observation of higher external voids with Well-Root PT under ultrasonic condensation, suggesting that its creamier consistency and prolonged ultrasonic activation (10 **s**) may have contributed to increased porosity, as seen in our results. The absence of dentinal moisture and anatomical variability in resin models may limit direct clinical applicability, emphasizing the need for validation in natural teeth.

Caries or traumatic injuries often cause pulp necrosis in permanent teeth. This arrests the root development, resulting in teeth with short roots, thin dentin walls, and a wide-open apex. The main challenge in treatment is the lack of apical constriction to support conventional root canal treatment. The creation of an apical plug in a tooth with a large apical opening was developed to facilitate closure of the root apex and promote hard tissue formation at the tooth’s apex [39]. This approach shortens treatment time and improves biocompatibility by enhancing interaction with periapical tissues and promoting cell proliferation and differentiation [39,40]. Limited visibility, the need for careful application of condensation pressure near the apical opening, and the irregularity of dentin walls in open apices may hinder the ability of the bioceramic materials to achieve a complete seal of the dentin surface [31]. The obturation material must exhibit stability, good adaptation, and firm retention to effectively seal the canal, thereby preventing the entry of oral fluids and microorganisms [40]. Premixed bioceramics such as NuSmile NeoPUTTY and Well-Root PT facilitate the treatment protocol compared to MTA. Therefore, these materials were used in the present study. The findings indicated that the formation of voids remained within low limits, irrespective of the type of obturation material and the technique employed.

Other micro-CT studies have also shown that none of the bioceramic materials or techniques for their placement lead to the creation of apical plugs without voids [38]. Rotary endodontic instruments are also recommended for better condensation of the materials [41]. In the present study, we focused on the XP-endo Shaper and compared its effect on the quality of the apical plug using manual and ultrasonic condensation. The results showed no difference in the percentage of internal voids regardless of the technique used. However, the lowest percentage of external voids was observed when using the XP-endo Shaper in combination with NeoPutty, and the difference with most groups was not statistically significant. This finding may be attributed to the serpentine and reverse motor motion of XPS, which allows for three-dimensional material distribution with denser obturation and enhanced sealing capability. These results do not coincide with those published in the specialized literature, where using this instrument to create an apical plug on a simulated immature tooth with a traditional endodontic cavity led to a less dense obturation [31]. The difference may be due to the use of different materials, such as MTA versus premixed bioceramic putties, in the present study, and the better flowability and adaptability of the premixed bioceramics compared to MTA. This suggests that the instrument is a suitable alternative to ultrasonic condensation for the creation of apical plugs in the treatment of immature permanent teeth with pulp necrosis. The total porosity of the materials used is generally low (often below 5%). However, even small volumes of open (external) porosity are clinically significant, as they can disrupt the apical seal and increase the risk of reinfection [38,42]. In the present study, the total voids were below 4%, consistent with data reported in the literature [31,38,41].

Density and porosity are critical parameters that significantly influence endodontic treatment. High porosity with large pore diameters facilitates the entry of microorganisms and compromises the three-dimensional sealing [43]. Porosity in tricalcium silicate cements is an intrinsic property resulting from the spaces between the unhydrated particles of the material, which initially fill with water during the hydration process. During hydration, these spaces are initially saturated with water. As hydration continues, the resulting products gradually occupy these gaps, thus reducing the overall porosity. This phenomenon is crucial for optimizing the sealing ability of the material and, therefore, the treatment outcome [43].

Several studies have reported that manual condensation resulted in better quality MTA fillings (denser, less porous, more homogeneous) compared to ultrasonic activation [26,44,45]. In the current study, condensing Well-Root PT with 10 s of indirect ultrasonic activation resulted in higher porosity than manual condensation. Other authors found similar data using MTA. They, therefore, recommended that ultrasonic activation be reduced to less than 8 s because more prolonged activation may result in air bubble retention due to excessive vibration force [30,46]. This would increase the risk of microleakage, bacterial invasion, and endodontic treatment failure, jeopardizing the healing process in the periapical area and the long-term retention of the tooth [47].

The present study found no significant differences in internal voids across all materials and techniques (*p* > 0.05, Table 4), with values ranging from 0.635% to 1.078%. For instance, a study by Biočanin et al. reported porosity in Biodentine retrograde filling ranging from 0.8% to 1.2%, consistent with the present study’s range [48]. The homogeneity in internal void formation across materials and techniques suggests that the intrinsic properties of calcium silicate-based cements, such as their ability to fill voids during hydration, dominate over application technique variations.

### Limitations

While the low-void percentages observed in this study highlight the efficacy of the tested materials and techniques, certain limitations must be considered when interpreting these findings. The use of 3D-printed plastic models, standardized to a 2 mm apical canal diameter, provided a controlled environment for evaluating void formation, as noted in prior studies using similar models for endodontic research [17]. The in vitro environment lacks dynamic biological responses that could affect the long-term sealing ability and clinical success of the tested materials. Furthermore, these models do not replicate the biological and anatomical complexities of natural immature teeth, such as dentinal tubule moisture and irregular canal morphology, which can influence material adaptation, sealing performance, and void formation in clinical settings. For instance, the higher external voids observed with Well-Root PT under ultrasonic condensation may be exacerbated in natural teeth due to moisture interactions, as calcium silicate-based materials rely on hydration for optimal setting [46]. This limitation suggests that our results may overestimate the sealing ability of these materials in vivo. Additionally, the study evaluated only three bioceramic materials (Biodentine, NuSmile NeoPUTTY, and Well-Root PT) and three application techniques (manual condensation, XP-endo Shaper, and ultrasonic condensation), which limits the generalizability of our conclusions to other materials or methods. For example, the superior performance of XP-endo Shaper with NeoPUTTY and Biodentine in reducing external voids may not extend to other rotary systems or bioceramics with different viscosities. These constraints highlight the need for broader investigations into additional materials, techniques, and clinical variables, such as operator experience or irrigation protocols, to better reflect the complexities of apexification in clinical practice.

Future research should focus on extending these findings by conducting studies in extracted natural immature teeth to account for biological factors, such as dentinal tubule moisture and irregular canal morphology, which may influence material adaptation and void formation. Exploring variations in condensation parameters, such as adjusting the duration and intensity of ultrasonic activation, could optimize material adaptation and minimize external voids. Additionally, assessing newer or alternative calcium silicate-based materials with different compositions or viscosities would provide insights into their impact on void formation and sealing efficacy. Incorporating operator variability by examining the influence of experience and technique consistency would further enhance the clinical relevance of these findings. These studies should continue to leverage micro-computed tomography for high-resolution analysis and consider longitudinal clinical outcomes to validate the long-term sealing ability and treatment success of these materials and techniques in immature teeth.

## 5. Conclusions

This in vitro study demonstrated that Biodentine, NuSmile NeoPUTTY, and Well-Root PT, when used to create apical plugs in 3D-printed simulated immature teeth, produce low levels of internal and external voids, with total voids not exceeding 4% of the material volume. The application technique—manual condensation, ultrasonic condensation, or use of a rotary file (XP-endo Shaper)—did not significantly affect internal void formation. However, external voids were notably higher with Well-Root PT under ultrasonic condensation, while XP-endo Shaper with NeoPUTTY and Biodentine resulted in the lowest external void percentages, suggesting its potential as an effective alternative to ultrasonic condensation. These findings indicate that the interaction between material and application technique plays a crucial role during the creation of apical plugs. The standardized 3D-printed models ensured consistent canal dimensions, facilitating reliable comparisons, but their in vitro nature limits direct clinical applicability due to the absence of biological and anatomical complexities, such as dentinal tubule moisture or variable canal morphology.

## Figures and Tables

**Table 1:** Materials, chemical composition, mixing method, and manufacturer of the cements.

Material	Manufacturer	Composition	Mixing Method
Biodentine XP^®^	Septodont, Saint-Maur-des-Fosses, France	Powder: Tricalcium silicate, dicalcium silicate, calcium silicate, iron oxide, zirconium oxide; Liquid: calcium chloride	Capsule of pre-dosed powder and liquid, mixing with high-speed mixer at 6200 rpm
Well-Root PT	Vericom Co., Republic of Korea	Calcium aluminosilicate, zirconium oxide, calcium aluminate, calcium sulfate, thickening agent	Premixed material in a compule (0.25 g)
NeoPUTTY	NuSmile Ltd., Houston, TX, USA	Tricalcium and dicalcium aluminosilicate, zirconium oxide, tantalum oxide, thickening agent	Premixed material in a syringe (0.5 g)

**Table 2:** Distribution of the study groups.

Group	Number of Samples	Material	Application Technique
Group 1	12	Biodentine XP^®^	Manual condensation—condensation of the material—was conducted with an endodontic plugger (1 mm tip diameter), selected to fit approximately 50% of the 2 mm apical canal diameter, ensuring controlled compaction.
Group 2	12		XP-endo Shaper: The condensation of the material was completed using the XP-endo Shaper (XPS) (FKG Dentaire SA, La Chaux-de-Fonds, Switzerland). The XPS file was positioned in the canal at 1 mm shorter than the working length (20 mm) and operated at 800 rpm, 1 N·cm in a counterclockwise motion [31]. After the specified working length was reached, the file was carefully retracted from the canal while maintaining its rotary motion.
Group 3	12		Indirect ultrasonic condensation—indirect ultrasonic activation at 25 kHz was applied to the material for 10 s by placing the shaft of an endodontic plugger (1 mm tip diameter) in contact with the ultrasonic tip, with the plugger sized to fit approximately 50% of the 2 mm apical canal diameter.
Group 4	12	Well-Root PT	Manual condensation
Group 5	12		Rotary file condensation (XP-endo Shaper)
Group 6	12		Indirect ultrasonic condensation
Group 7	12	NeoPUTTY	Manual condensation
Group 8	12		Rotary file condensation (XP-endo Shaper)
Group 9	12		Indirect ultrasonic condensation

**Table 3:** Two-way ANOVA analysis of the effect of materials, application technique, and their interaction on internal, external, and total void formation.

Dependent Variable	Source	F-Value	*p*-Value
Internal voids	Material	0.700	0.499
	Technique	1.205	0.304
	Interaction	2.717	0.034
External voids	Material	27.266	<0.001
	Technique	11.365	<0.001
	Interaction	4.169	0.004
Total voids	Material	14.567	<0.001
	Technique	7.890	<0.001
	Interaction	3.456	0.012

**Table 4:** Pairwise comparison of the mean ± standard deviation of the percentage of formed voids depending on the apical plug material and technique used. Different superscript uppercase letters (row) or lowercase letters (column) indicate significant differences (*p* < 0.05). If data share the same uppercase letter in a row or lowercase letter in a column, there is no significant difference between them (*p* < 0.05).

Technique				
**Materials**		Manual condensation	Ultrasonic condensation	Rotary file (XP-endo Shaper) condensation
		Internal voids (%)		
	Biodentine	0.64 ± 0.36 ^A,a^	0.80 ± 0.51 ^A,a^	1.08 ± 0.55 ^A,a^
	NeoPutty	0.69 ± 0.27 ^A,a^	0.84 ± 0.13 ^A,a^	0.78 ± 0.20 ^A,a^
	WellRoot PT	0.93 ± 0.41 ^A,a^	0.92 ± 0.19 ^A,a^	0.74 ± 0.24 ^A,a^
		External voids (%)		
	Biodentine	0.83 ± 0.67 ^A,a^	1.28 ± 0.74 ^A,a^	0.65 ± 0.84 ^A,ab^
	NeoPutty	1.37 ± 0.57 ^A,a^	1.12 ± 0.78 ^A,a^	0.38 ± 0.16 ^A,a^
	WellRoot PT	1.53 ± 0.71 ^A,a^	3.01 ± 1.30 ^B,b^	1.79 ± 0.72 ^A,b^
		Total voids (%)		
	Biodentine	1.46 ± 1.02 ^A,a^	2.06 ± 1.06 ^A,a^	1.72 ± 1.04 ^A,ab^
	NeoPutty	2.05 ± 0.63 ^A,a^	1.95 ± 0.76 ^A,a^	1.15 ± 0.53 ^A,a^
	WellRoot PT	2.44 ± 0.60 ^A,a^	3.90 ± 1.42 ^B,b^	2.52 ± 0.62 ^A,b^

## Data Availability

The original contributions presented in this study are included in this article, and further inquiries can be directed to the corresponding author.

## Abbreviations

The following abbreviations are used in this manuscript:

MTA	Mineral Trioxide Aggregate
Micro-CT	Micro-Computed Tomography
